# Anti-CXCR4 Single-Chain Variable Fragment Antibodies Have Anti-Tumor Activity

**DOI:** 10.3389/fonc.2020.571194

**Published:** 2020-12-18

**Authors:** Guang-Quan Liang, Jing Liu, Xiao-Xin Zhou, Ze-Xiong Lin, Tao Chen, Guo Chen, Henry Wei

**Affiliations:** ^1^ Department of Cell Biology and Institute of Biomedicine, Guangdong Provincial Key Laboratory of Bioengineering Medicine, Guangdong Provincial Biotechnology Drug and Engineering Technology Research Center, National Engineering Research Center of Genetic Medicine, College of Life Science and Technology, Jinan University, Guangzhou, China; ^2^ Department of Medical Biochemistry and Molecular Biology, School of Medicine, Jinan University, Guangzhou, China

**Keywords:** CXCR4, scFv, cancer, antibody, yeast two-hybrid

## Abstract

Monoclonal antibodies (mAbs) are large and have limitations as cancer therapeutics. Human single-chain variable fragment (scFv) is a small antibody as a good alternative. It can easily enter cancer tissues, has no immunogenicity and can be produced in bacteria to decrease the cost. The chemokine receptor CXCR4 is overexpressed in different cancer cells. It plays an important role in tumor growth and metastasis. Its overexpression is associated with poor prognosis in cancer patients and is regarded as an attractive target for cancer treatment. In this study, a peptide on the CXCR4 extracellular loop 2 (ECL2) was used as an antigen for screening a human scFv antibody library by yeast two-hybrid method. Three anti-CXCR4 scFv antibodies were isolated. They could bind to CXCR4 protein and three cancer cell lines (DU145, PC3, and MDA-MB-231) and not to 293T and 3T3 cells as negative controls. These three scFvs could decrease the proliferation, migration, and invasion of these cancer cells and promote their apoptosis. The two scFvs were further examined in a mouse xenograft model, and they inhibited the tumor growth. Tumor immunohistochemistry also demonstrated that the two scFvs decreased cancer cell proliferation and tumor angiogenesis and increased their apoptosis. These results show that these anti-CXCR4 scFvs can decrease cancer cell proliferation and inhibit tumor growth in mice, and may provide therapy for various cancers.

## Introduction

Chemokines belong to a large family of soluble chemotactic cytokines that bind to G protein-coupled receptors (GPCRs) to cause a cellular response, such as cell migration and adhesion (1). They can be divided into inflammatory and homeostatic chemokines according to their functions. About 50 human chemokines and nearly 20 their receptors have been identified and characterized since the discovery of the first chemotactic cytokine interleukin 8 (IL-8) (2, 3). Chemokines and their receptors play an important role in inflammation, infection, cardiovascular disease and cancer development. The chemokine receptor CXCR4 is a GPCR and consists of 352 amino acids (4). It is a good drug target for different cancers. It is over-expressed in different cancers including ovarian (5), prostate (6) and lung cancers (7). CXCR4 overexpression is correlated with poor prognosis in cancer therapy. Numerous clinical studies showed that patients with high CXCR4-expressing tumors had significantly decreased survival rate in lung cancer (8), acute myeloid leukemia (9) and renal cell carcinoma (10). High CXCR4 expression was correlated with poor response to sunitinib (a chemotherapy drug) for patients with metastatic renal cancer (10, 11). CXCR4 can bind to its ligand CXCL12 (also known as stromal cell-derived factor 1, SDF-1) (12). Studies with a neuroblastoma or prostate tumor mouse model showed that high CXCL12 expression in the tumor cells recruited CXCR4-positive monocytes and stimulated the formation of new tumor blood vessels (13, 14). Furthermore, tissues with high CXCL12 expression, such as lymph nodes, liver and lung, are the most common cancer metastatic sites, and cancer cells migrate to these tissues in a CXCL12-dependent manner (15). CXCR4/CXCL12 axis is involved in tumor progression. The activation of CXCR4 by CXCL12 mediates tumor cell survival and proliferation and enhances primary tumor progression, angiogenesis and metastasis (16). Therefore, CXCR4/CXCL12 axis is a significant target for tumor therapy.

Targeted therapy becomes a major cancer treatment due to its specific anti-cancer effect and less side effect (17). CXCR4 antagonists are in different development stages. The small-molecule CXCR4 antagonist Plerixafor (also named as AMD3100) was approved by FDA for hematopoietic stem cell mobilization as an injectable agent, and it can block CXCR4 activation to inhibit metastasis of multiple solid tumors (16, 18). Monoclonal antibodies (mAbs) are attractive medicines for cancer treatment (19). Ulocuplumab (BMS-936564) is a mAb against CXCR4, and is currently in phase Ib/II study to determine its safety and tolerability for patients with relapsed/refractory multiple myeloma (20). F50067, a humanized mAb targeting CXCR4, demonstrated preclinical anti-tumor activity in multiple myeloma, and phase I study was performed to assess the safety and efficacy of F50067 alone and in combination with lenalidomide and dexamethasone in relapsed or refractory multiple myeloma (21). However, mAb has the following major disadvantages: it is difficult in penetrating tumor tissues due to its large size (150 kDa). It contains antigenicity because it is commonly isolated in mice, and the mouse mAb is recognized as a foreign antigen by human. It is expensive to be manufactured because it needs to be expressed in mammalian cells. Therefore, the low molecular weight antibody fragments, such as single-chain variable fragment (scFv), are regarded as good alternatives for conventional mAbs. scFv has the following advantages compared with mAb (22): it can easily penetrate into the tumor tissue due to its small molecular weight. It contains no antigenicity if it is isolated from a human scFv library. It can be produced in bacteria, thereby greatly reducing the manufacturing cost (23, 24). scFv does not contain Fc domain of mAb so that it cannot mediate antibody dependent cell-mediated cytotoxicity (ADCC), complement-dependent cytotoxicity (CDC), and antibody-mediated opsonization. Lack of Fc can decrease its efficacy for cancer therapy, but scFv can be modified in different ways to increase its efficacy for therapeutic use as described below.

scFv antibody comprises a variable region of heavy chain and a variable region of light chain, and the two chains are connected by a short peptide linker (25). It can act as an antagonist to block tumor growth signaling, induce tumor cell apoptosis and suppress tumor growth (22). scFv antibody can be modified to become a bivalent antibody by combining two different scFvs targeting two different target proteins. It can also be an immune conjugate by fusing a tumor-targeting scFv with a toxin molecule (26). scFvs may become major therapeutics for targeted cancer therapy.

In this study, the CXCR4 extracellular loop 2 (ECL2) was selected as an antigen for screening a human scFv antibody library. Three human anti-CXCR4 scFvs were isolated from a human scFv library by yeast two-hybrid technology (27). They were expressed in *E. coli*, purified and refolded. The binding of these three anti-CXCR4 scFvs to CXCR4 was confirmed. They could inhibit cancer cell proliferation, migration and invasion and promote the cancer cell apoptosis *in vitro*. Two of these three scFvs were further tested for their anti-tumor effect *in vivo*, and they could inhibit the tumor growth in a prostate cancer xenograft mouse model. These anti-CXCR4 scFvs may become potential anti-cancer therapeutics for the treatment of different cancers.

## Materials and Methods

### Materials

Anti-His antibody-HRP was purchased from Thermo Fisher (Waltham, MA, USA). Saccharomyces cerevisiae strain AH109, YPD medium and yeast selective culture medium were purchased from Clontech (Palo Alto, CA, USA). T4 DNA ligase and restriction enzymes were purchased from Takara Biomedicals (Kyoto, Japan). pGBKT7-CXCR4 was constructed and optimized by Genewiz (Suzhou, China). *E. coli* strains (DH5a, BL21) and Ni-MAC Cartridge were from Novagen. Primary antibodies against Bax, Bcl-2, cleaved caspase-8 (c-caspase-8), cleaved caspase-3 (c-caspase-3), cleaved-PARP-1 (c-PARP-1), HRP-conjugated secondary antibody, and Alexa Fluor 488-labeled secondary antibody were from Beyotime Biotechnology (Shanghai, China). Primary antibody against pro-caspase-9 was from ZFdows BIO (Nanjing, China). Primary antibody against p-p53 and secondary antibody m-IgGκ BP-HRP were from Santa Cruz Biotechnology (CA, USA). Primary antibody against β-actin was from ZSGB-BIO (Beijing, China).

### Assays for the Bait Plasmid Autonomous Activation

To examine the bait plasmid autonomous activation, yeast competent cell AH109 was prepared and transformed with bait plasmid (pGBKT7-CXCR4). The transformed AH109 was plated on the following media: SD/-Trp, SD/-Trp/-His, or SD/-Trp/-Ade. These plates were incubated at 30°C for 3-6 days and observed whether the colonies could grow.

### The Human scFv Library Screening

The human scFv library was constructed by referring to the previous reports (28–30). Briefly, human PBMCs were isolated using Ficoll Paque Plus (Amersham Pharmacia Biotech, Piscataway, NJ, USA) according to the manufacturer’s instructions. mRNA was extracted from PBMCs using the Dynabeads mRNA direct kit (Invitrogen) and was used to synthesize full-length cDNA with the SMART cDNA library construction kit (Clontech). The variable regions of human immunoglobulin (Ig) heavy-chains (VH) and light-chains (VL) were amplified by polymerase chain reactions (PCRs) using a set of primers as previously described (28, 29), and the VH and VL gene repertoires were linked by overlapping extension PCR. The scFv fragments were cloned into yeast vector to generate the human scFv library.

After yeast AH109 competent cells were transformed with the pGBKT7-CXCR4 plasmid, they were also transformed with the human scFv library. They were incubated on SD/-Trp/-Leu/-His + 10 mM 3-AT plates. The colonies which grew on the plates were randomly picked and re-streaked on SD/-Trp/-Leu/-His/-Ade + 10 mM 3-AT plates and incubated at 30°C for 4–6 days to confirm the positive colonies. The candidate positive colonies were transferred to SD/-Trp/-Leu/-His/-Ade liquid medium and cultured at 30°C at 250 rpm. The plasmid DNA was isolated from each colony using the yeast plasmid isolation kit (Clontech). Each colony plasmid DNA was transformed into AH109 containing empty vector pGBKT7, bait vector pGBKT7-CXCR4, or the vectors containing the control antigens. Each anti-CXCR4 scFv antibody clone was DNA-sequenced, and the DNA sequence from each clone was compared with each other to identify the same clones.

### Purification and Refolding of scFv Antibody Proteins

Each anti-CXCR4 scFv gene was cloned in pET-28a-sumo vector containing His tag. The plasmid DNA was transformed into BL21. Each colony was cultured in 4 ml LB including 50 μg/ml of Kanamycin. Then, bacteria were cultured in 400 ml LB including 50 μg/ml of Kanamycin until the optical density reached OD600 0.6-0.8. Bacteria were cultured with IPTG (0.5 mM) for 6 h at 37°C, 30°C, 25°C or 18°C. The total bacterium proteins were analyzed by 12% SDS-PAGE.

Following the IPTG induction, the bacteria were lysed by sonication. After the centrifugation at 13,000 g for 30 min at 4°C, the bacteria were washed twice with the buffer containing 50 mM Tris-HCl (pH 8.0), 150 mM NaCl and 2% Triton X-100 for 2 h each time at room temperature. The inclusion bodies were solubilized in the buffer containing 50 mM Tris–HCl (pH 8.0), 8 M urea, 0.5 M NaCl and 10 mM β-mercaptoethanol for 2.5 h at room temperature, and centrifuged at 18,000 g for 30 min at 4°C to remove insoluble materials. The supernatant was filtered through 0.22 μm filter (Millipore, Billerica, MA, USA) to remove any remaining insoluble materials. The scFv proteins were purified by a Ni-MAC Cartridge (Novagen) under denaturing conditions (8 M urea). The purified scFv proteins were analyzed by SDS–PAGE.

Refolding of the scFv proteins was carried out by the stepwise dialysis method, which included dialysis against the buffer (3 mM GSH, 1 mM GSSG and 150 mM NaCl) containing urea at 8, 4, 2, 1, 0.5, 0.25, or 0 M. The refolded proteins were dialyzed against Tri-HCl (pH 9.0) and centrifuged at 13,000 g for 30 min to remove the insoluble materials. The refolded scFv proteins were analyzed by SDS–PAGE under non-reducing conditions and the protein concentrations were determined by the Bradford method.

### Western Blot Analysis

For Western blot analysis of anti-CXCR4 scFv protein expression, the total bacterial proteins were separated on a 12% SDS-PAGE and transferred to a PVDF membrane. After the membrane was blocked for 60 min at room temperature in the blocking buffer (PBS containing 0.5% Tween-20 and 5% nonfat dry milk powder), it was kept with anti-His 6 mAb overnight at 4°C. After washing, the membrane was kept with HRP-conjugated goat anti-mouse IgG (1/10,000) for 60 min at room temperature. The bands were visualized with ECL Western blot detection system.

To detect the expression of the apoptosis-related proteins Bax, Bcl-2, p-p53, pro-caspase-9, c-caspase-8, and c-caspase-3, Western blot analysis was performed as described above. Cells were incubated for 48 h with the scFv proteins or negative control antibodies (75 μg/ml) or PBS as a control. The membranes were incubated with the primary antibodies and HRP-conjugated secondary antibodies. β-actin was used as an internal control. The images were analyzed with ImageJ software (NIH, Bethesda, MD).

### Binding of the Anti-CXCR4 scFvs to CXCR4 Protein Was Detected By ELISA

A 96-well plate was incubated with the CXCR4 protein (0.2 µg each well) purchased commercially (Shanghai Bootech BioSci. and Technol., Shanghai, China). After washing with PBS, it was treated with BSA. Solution was removed, and it was treated for 1 h at 37°C with the purified anti-CXCR4 scFvs. After washing with PBS, it was treated for 1 h at room temperature with anti-His-HRP at 1:1,000 dilution. After it was washed, 100 μl TMB was added. Then, 50 μl of 1 M sulfuric acid was added. The optical density was detected.

### Binding of the Anti-CXCR4 scFvs to Cells Was Detected by Flow Cytometry

Cells (1×10^6^/ml) were incubated on ice for 1 h with the test reagents. After washing cells for three times, anti-CXCR4 scFvs, negative control antibodies or no antibody control were examined by PE-conjugated anti-His 6 antibody. Anti-CXCR4 antibody (positive control) or isotype control antibody were examined by PE-conjugated mouse IgG kappa binding protein. Cells were examined with FACS Calibur. FlowJo was used to examine the data.

### Cell Proliferation Assay

Cancer cells (DU145, PC3, and MDA-MB-231, ATCC) were incubated overnight in culture medium containing 10% FBS. Following the starvation, cells were washed and cultured for three days with serum-free medium containing CXCL12 (100 ng/ml, Sino Biological) and the anti-CXCR4 scFvs. They were cultured with MTT (Sigma) at 37°C for 4 h. After removing culture medium, DMSO was included, and optical density was detected.

### Detection of Apoptosis by Flow Cytometry

Cells were incubated overnight. After the starvation for 4 h in serum-free culture medium, CXCL12 (100 ng/ml) and the anti-CXCR4 scFvs, the negative control antibodies (50 µg/ml) or PBS as a control were added to cells, and cells were cultured for two days at 37°C. They were cultured for 15 min in dark with Annexin-V/FITC and PI (Sangon Biotech). Flow cytometer FACS Calibur was used to detect the apoptosis, and the results were examined with the FlowJo.

Expression of apoptosis-related proteins Bax, Bcl-2, c-caspase-3, and c-PARP-1 was detected by flow cytometry as described above. The three cancer cell lines were treated for 48 h with the anti-CXCR4 scFvs, the negative control antibodies (75 μg/ml), or PBS as a control. Cells were incubated for 1 h with primary antibodies and for 1 h with Alexa Fluor 488-labeled secondary antibody.

### Wound Healing Assay

Cells (DU145, PC3, and MDA-MB-231) were incubated in a 12-well plate in culture medium containing 10% FBS. After the starvation for 4 h, cell monolayers were scratched. Cells were incubated in culture medium supplemented with 1% FBS, CXCL12 (100 ng/ml) and the anti-CXCR4 scFvs or negative control antibodies. Images were taken at 0 and 24 h after the cell wounds were made. The wound widths were determined by Image-Pro Plus 6.0. The migration percentages were determined: Lm = (L0-Lt)/L0 × 100%. Lm is the migration percentage, L0 is wound width at 0 h, and Lt is wound width at 24 h.

### Transwell Assay

For the detection of cell migration, cells in culture medium including 1% FBS were placed to each transwell (BD Biosciences) that was put in a 24-well plate. Each well was filled with culture medium including 20% FBS. Cells were incubated for one day with CXCL12 (100 ng/ml) and the anti-CXCR4 scFvs or negative control antibodies. Cells in the upper chamber were gently scraped off. The cells that crossed over the membrane were treated with crystal violet, and images were photographed. Crystal violet of stained cells was solubilized in acetic acid. Optical density was detected. For the detection of cell invasion, matrigel was placed in transwells, and then, cells were added. The other steps are the same as described above.

### Xenograft Mouse Model

The animal study protocols were approved by the Institutional Animal Care and Use Committee of Jinan University. Four-week-old BALB/c nude mice were from Beijing HuaFuKang Bioscience. Six-week-old mice were inoculated with DU145 cells subcutaneously into the right flank. After tumors were about 100 mm^3^, mice were randomized to the different groups. The anti-CXCR4 scFvs, negative control antibody (Her2-13C1) (10 mg/kg) or DDP (2 mg/kg) were administrated by intravenous inoculation. The tumor volume (V) was determined: V= (length × width^2^)/2.

### Immunohistochemical Staining

After euthanization of the mice on day 24 after the first inoculation, the tumors were excised. Paraffin blocks were prepared and sectioned. The tissue sections were incubated with HE, or antibodies against Ki67, CD31, and c-caspase-3. After washing, they were treated with goat anti-rat antibody conjugated with HRP. After washing, they were treated with DAB chromogen. The images were taken. Image-Pro Plus was used to analyze the images for IOD (integrated optical density).

### Statistics

GraphPad Prism was used to make the images. One-way analysis of variance test (one-way ANOVA) was used for data analysis. The results represent mean ± SD. *P* < 0.05 indicates statistically significant.

## Results

### Identification of the Positive Anti-CXCR4 scFv Clones

**Figure 1:**
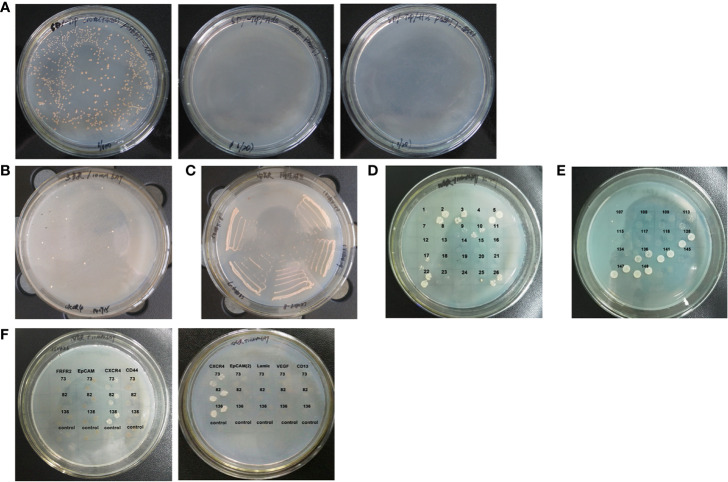
Cloning anti-CXCR4 scFvs from human scFv antibody library. **(A)** Yeast strain AH109 competent cells were transformed with the bait plasmid pGBKT7-CXCR4 and cultured on the different plates. The clones grew on SD/-Trp plate (Left), but not on SD/-Trp/-Ade plate (Middle) and SD/-Trp/-His plate (Right). These results showed that the bait plasmid alone did not activate the expression of the reporter genes. **(B)** A total of 241 candidate positive scFv clones were obtained from screening the scFv library. The image shows some representative clones of the 241 candidate positive clones which grew on SD/-Trp/-Leu/-His plates containing 10 mM 3-AT. **(C)** The image shows some representative clones of the 241 candidate positive scFv clones which were re-streaked on SD/-Trp/-Leu/-His/-Ade plates containing 10 mM 3-AT, so that the clones which grew on the plate were the candidate positive scFv clones, and the false positive clones did not grow. A total of 221 candidate positive scFv clones were identified. **(D)** The image shows some representative clones of the 221 candidate positive scFv clones from which the plasmid DNA was isolated and transformed into AH109 containing empty vector pGBKT7. The clones which grew on SD/-Trp/-Leu/-His/-Ade plates containing 10 mM 3-AT were false positive clones, and the candidate positive clones did not grow. A total of 85 candidate positive scFv clones were obtained. **(E)** The image shows some representative clones of the 85 candidate positive scFv clones from which the plasmid DNA was isolated and transformed into AH109 containing bait plasmid pGBKT7-CXCR4, which were cultured on the SD/-Trp/-Leu/-His/-Ade plates containing 10 mM 3-AT. A total of 14 candidate positive scFv clones were obtained. **(F)** The images show some representative clones of the 14 positive clones from which the plasmid DNA was isolated and transformed into AH109 containing bait plasmid pGBKT7-CXCR4 or the unrelated antigens as negative controls. Three positive clones contained scFvs which could interact with CXCR4, but, not with the unrelated antigens.

The bait plasmid pGBKT7-CXCR4 was transformed into yeast strain AH109 to detect the autonomous activation. Result showed that AH109 transformed with pGBKT7-CXCR4 could grow on SD/-Trp plate, but, not on SD/-Trp/-Ade and SD/-Trp/-His plates (**Figure 1A**). This result proved that the bait plasmid did not activate Ade and His reporter genes. Furthermore, AH109 transformed with pGBKT7-CXCR4 grew at the same rate as AH109 transformed with the empty vector. Therefore, the bait plasmid was suitable for yeast two-hybrid screening.

Bait plasmid pGBKT7-CXCR4 was transformed into yeast AH109 competent cells, into which the human scFv antibody library was subsequently also transformed. A total of 241 candidate positive scFv clones grew on the SD/-Trp/-Leu/-His plates containing 10 mM 3-AT (**Figure 1B**). These clones were re-streaked in SD/-Trp/-Leu/-His/-Ade plates containing 10 mM 3-AT, and 221 candidate positive scFv clones were obtained (**Figure 1C**). In order to eliminate the false positive clones, plasmid DNA of these clones was transformed into AH109 containing empty vector pGBKT7, which was cultured on SD/-Trp/-Leu/-His/-Ade plates containing 10 mM 3-AT. The clones which grew on the plates were false positive clones. A total of 85 candidate positive scFv clones that did not grow on the plates were obtained (**Figure 1D**). Plasmid DNA of these clones was transformed into AH109 containing bait plasmid pGBKT7-CXCR4, and 14 candidate positive scFv clones grew on the SD/-Trp/-Leu/-His/-Ade plates containing 10 mM 3-AT (**Figure 1E**). Plasmid DNA of these clones was transformed into AH109 containing vectors with the unrelated antigens to determine the specificity of these clones. Only three positive scFv clones specifically bound to bait plasmid pGBKT7-CXCR4, not to the other unrelated antigens (**Figure 1F**).

These three scFv clones were DNA-sequenced, and analyzed by DNAMAN software. Three different anti-CXCR4 scFvs were identified and named aCX73, aCX82, and aCX136. Their DNA sequences were submitted to European Nucleotide Archive under accession number LR743525, LR743526 and LR743527, respectively. These three scFv DNA sequences were translated to amino acid sequences, which were compared. Amino acid sequence alignment of the three scFvs demonstrated that the CDR regions of their antibody heavy chains and light chains were different from each other.

### Expression, Purification, and Refolding of scFv Proteins From Inclusion Bodies

**Figure 2:**
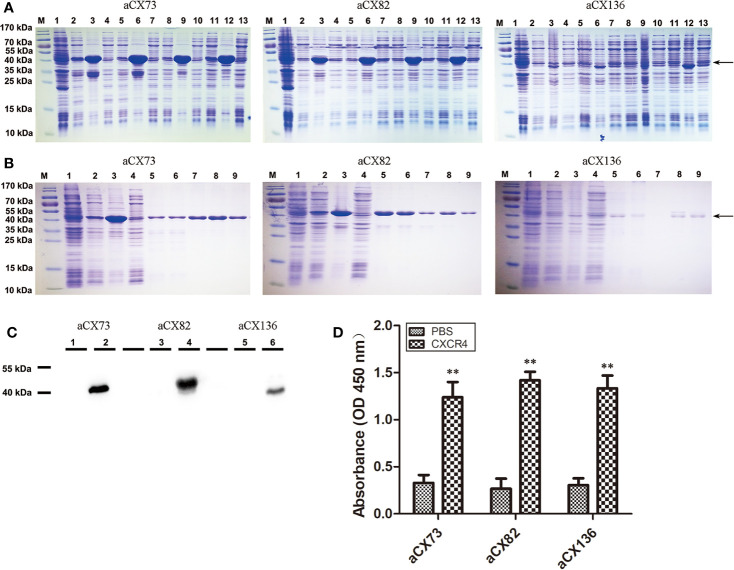
Purification and analysis of the three anti-CXCR4 scFvs. **(A)** Detection of the expression of three anti-CXCR4 scFvs (aCX73, aCX82 and aCX136) by SDS-PAGE. M, marker; 1, total proteins of un-induced bacteria; 2–4, 5–7, 8–10, and 11–13, proteins of bacteria induced for 6 h with 0.5 mM IPTG at 37°C, 30°C, 25°C, and 18°C, respectively; 2, 5, 8 and 11, total proteins of induced bacteria; 3, 6, 9, and 12, insoluble portion of sonicated induced bacterium lysate; 4, 7, 10, and 13, soluble supernatant of sonicated induced bacterium lysate. **(B)** scFv purification and refolding. M: marker; 1, total proteins of un-induced bacteria; 2–4, proteins of bacteria induced for 6 h with 0.5 mM IPTG at 30°C; 2, total proteins of induced bacteria; 3, insoluble portion of sonicated induced bacterium lysate; 4, soluble supernatant of sonicated induced bacterium lysate; 5, solubilized inclusion bodies; 6, flow-through portion; 7, eluted portion with 0 mM imidazole; 8, eluted portion with 250 mM imidazole; 9, refolded scFvs. **(C)** The expression of the scFvs was confirmed by Western blotting with a mouse anti-His antibody. 1, 3, and 5, total proteins of un-induced bacteria; 2, 4, and 6, total proteins of induced bacteria. **(D)** ELISA was performed to detect the binding of the three purified anti-CXCR4 scFvs to the CXCR4 protein purchased. The results represent mean ± S.D (n=5). **P < 0.01.

The three scFvs were cloned to pET-28a-sumo expression vector. The recombinant plasmids were transformed into *E. coli*, and induced by 0.5 mM IPTG at 37°C, 30°C, 25°C, or 18°C. The results showed that anti-CXCR4 scFv fusion proteins were over-expressed as inclusion bodies (**Figure 2A**).

Inclusion bodies of scFvs were obtained by cells sonication, centrifugation and solubilization in 8 M urea. His-tagged scFvs in inclusion bodies were purified under denatured condition using nickel-chelating His-trap column. Each purified scFv showed a single band (**Figure 2B**), and these denatured scFvs were used for further refolding experiments.

In order to optimize the refolding conditions, an oxido-shuffling system was used by adding 1 mM GSSG and 3 mM GSH to the refolding buffer at different urea concentrations. The purity of the refolded scFvs was estimated by 12% SDS-PAGE. The predicted molecular mass of aCX73, aCX82 and aCX136 were 43.2 kDa, 42 kDa, and 40 kDa, respectively, and were confirmed by Western blot analysis (**Figure 2C**).

### The Anti-CXCR4 scFvs Can Bind to CXCR4 Protein and Cancer Cells

ELISA was performed to detect the binding of the three anti-CXCR4 scFvs to CXCR4 protein. The data showed that the three scFvs could bind to CXCR4 (**Figure 2D**).

**Figure 3:**
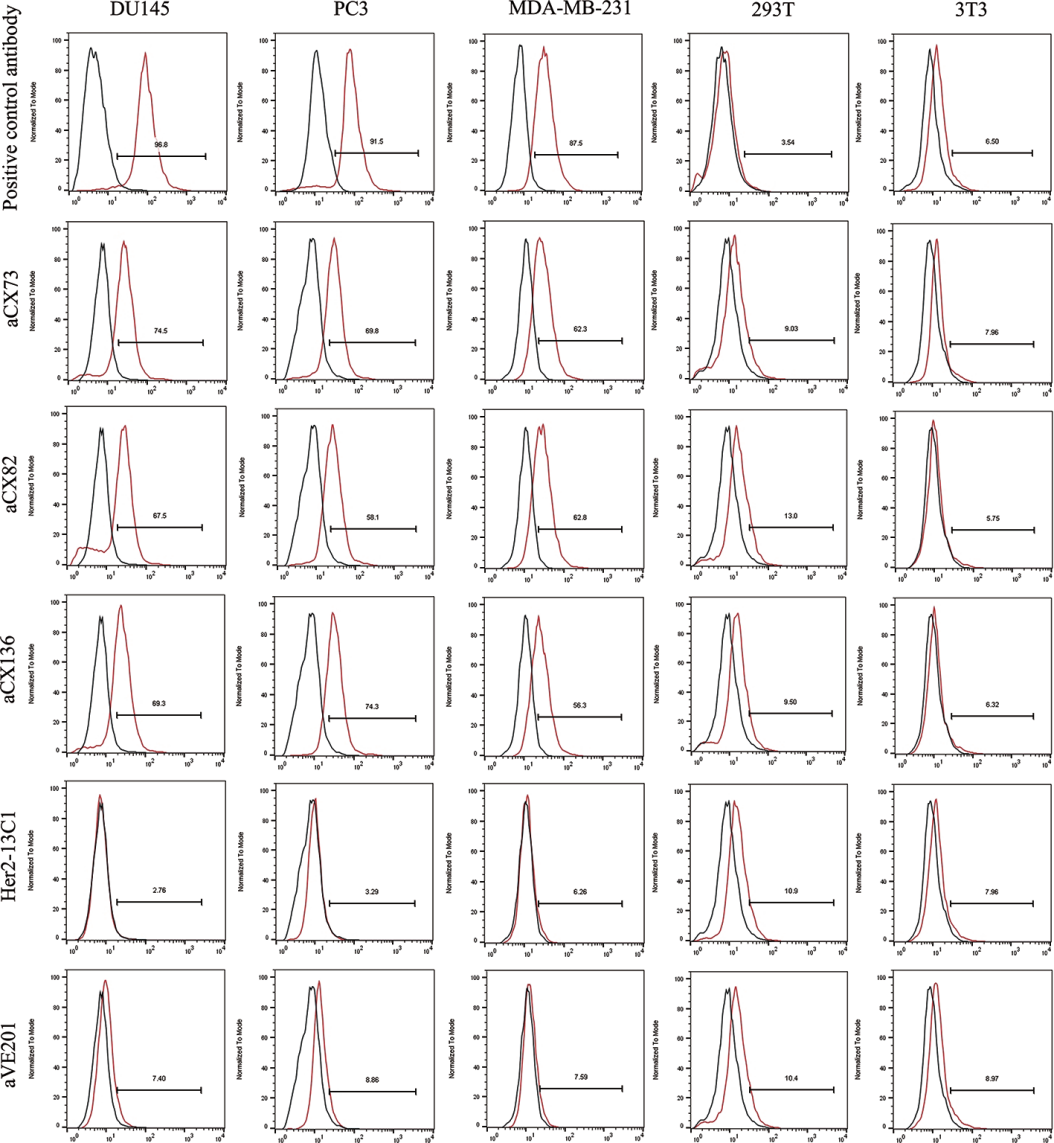
The binding of the three anti-CXCR4 scFvs to cancer cells (DU145, PC3 and MDA-MB-231) was tested by flow cytometry. 293T and 3T3 cells were tested as controls. Binding of an isotype control antibody or no antibody control was shown as black curves. Binding of the three anti-CXCR4 scFvs, anti-CXCR4 positive control antibody or the negative control antibodies (Her2-13C1 and aVE201) was shown as red curves.

Flow cytometry analysis was performed to check whether the three anti-CXCR4 scFvs could bind to cancer cells. The three scFvs and the anti-CXCR4 antibody as the positive control could bind to the three cancer cell lines (DU145, PC3, and MDA-MB-231), and have no or low binding to 293T and 3T3 as control cells. Two negative control antibodies (Her2-13C1 and aVE201) showed no or low binding to these cells (**Figure 3**). These two negative control antibodies were cloned previously in our group in a separate study (previously unpublished data) and could not bind to CXCR4.

For DU145 cells, percentages of aCX73, aCX82 and aCX136 positive cells were 74.5% (median fluorescence intensities (MFI) 30.4), 67.5% (MFI 30.5) and 69.3% (MFI 29.4) respectively. Conversely, only 2.76% (MFI 25.4) and 7.4% cells (MFI 23.6) were stained positive for Her2-13C1 and aVE201 (controls). For PC3 cells, percentages of aCX73, aCX82, and aCX136 positive cells were 69.8% (MFI 35.8), 58.1% (MFI 34.4), and 74.3% (MFI 36.7), respectively. Conversely, only 3.29% (MFI 30.2) and 8.86% cells (MFI 29.3) were stained positive for Her2-13C1 and aVE201 (controls). For MDA-MB-231 cells, percentages of aCX73, aCX82, and aCX136 positive cells were 62.3% (MFI 35.1), 62.8% (MFI 34.9), and 56.3% (MFI 33.7), respectively. Conversely, only 6.26% (MFI 28.5) and 7.59% cells (MFI 27.8) were stained positive for Her2-13C1 and aVE201 (controls). For the control cells (293T and 3T3), similar percentages and MFIs were detected for the three scFvs compared to the control antibodies (**Figure 3**).

### The Anti-CXCR4 scFvs Decrease Cancer Cell Proliferation

**Figure 4:**
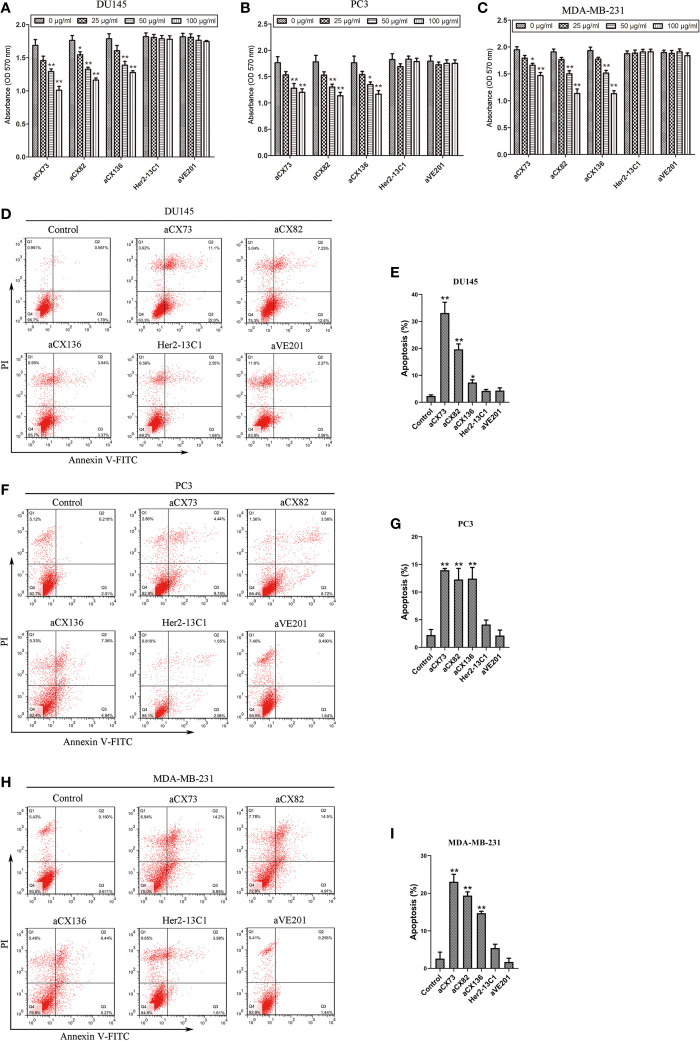
Anti-CXCR4 scFvs can decrease cell multiplication and increase apoptosis. Cell multiplication was examined to detect the inhibitory effect of three anti-CXCR4 scFvs (aCX73, aCX82 and aCX136) on the multiplication of DU145 **(A)**, PC3 **(B),** and MDA-MB-231 **(C)**. Cells were incubated for three days with CXCL12 (100 ng/ml) and the three anti-CXCR4 scFvs or negative control antibodies (Her2-13C1 and aVE201). Detection of apoptosis was carried out for DU145 **(D, E)**, PC3 **(F, G)** and MDA-MB-231 **(H, I)**. Cells were cultured for two days with the CXCL12 (100 ng/ml) and three anti-CXCR4 scFvs or negative control antibodies (Her2-13C1 and aVE201) (50 µg/ml). Apoptosis was detected by Annexin V-FITC and PI. The results represent mean ± S.D (n=5). *P < 0.05 and **P < 0.01.

Cell proliferation assay was performed to examine the effect of the three scFvs on the cancer cell proliferation. Data showed that the three scFvs decreased the proliferation of the three cancer cell lines (**Figures 4A–C**). Two negative control antibodies (Her2-13C1 and aVE201) did not decrease the cell proliferation. Percentages of growth inhibition were determined when cells were treated with the scFvs (100 µg/ml) compared to no scFv treatment controls. For DU145 cells, percentages of growth inhibition were 40.59% (aCX73), 33.90% (aCX82), and 30.00% (aCX136). For PC3 cells, percentages of growth inhibition were 31.82% (aCX73), 37.22% (aCX82), and 34.64% (aCX136). For MDA-MB-231 cells, percentages of growth inhibition were 24.62% (aCX73), 39.79% (aCX82), and 40.82% (aCX136) (**Figures 4A–C**). These results suggested that the three scFvs could inhibit the proliferation of these three cancer cell lines.

### Induction of Cancer Cell Apoptosis by scFvs Was Detected by Flow Cytometry

The percentages of apoptotic cells were analyzed by flow cytometry with Annexin V-FITC and PI staining after DU145, PC3, and MDA-MB-231 cells were treated with different antibodies. The three scFvs induced apoptosis in DU145, PC3, and MDA-MB-231 compared with the PBS control (**Figures 4D–I**). In contrast, the two negative control antibodies (Her2-13C1 and aVE201) did not increase apoptosis in these cells.

**Figure 5:**
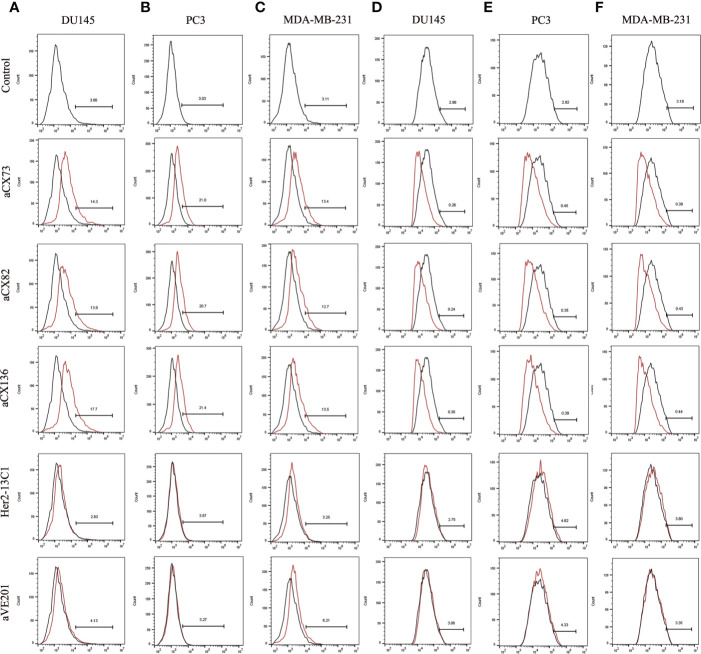
Expression of the apoptosis-related proteins Bax and Bcl-2 was detected by flow cytometry. Bax expression was examined after DU145 **(A)**, PC3 **(B)**, and MDA-MB-231 **(C)** were treated for 48 h with the three anti-CXCR4 scFvs (aCX73, aCX82, and aCX136). Bcl-2 expression was also tested in DU145 **(D)**, PC3 **(E)**, and MDA-MB-231 **(F)**. The PBS control and the two negative control antibodies (Her2-13C1 and aVE201) were also included.

**Figure 6:**
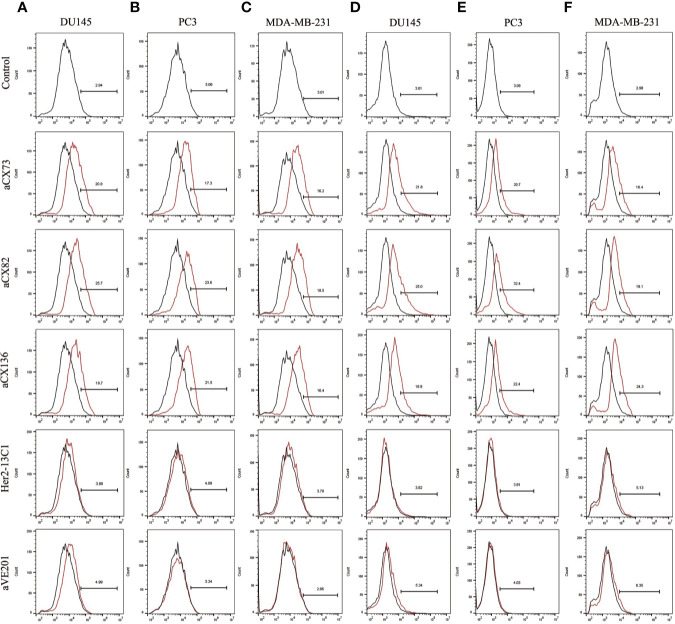
Expression of the apoptosis-related proteins c-caspase-3 and c-PARP-1 was also detected by flow cytometry. C-caspase-3 expression was examined after DU145 **(A)**, PC3 **(B)**, and MDA-MB-231 **(C)** were treated for 48 h with the three anti-CXCR4 scFvs (aCX73, aCX82 and aCX136). C-PARP-1 expression was also tested in DU145 **(D)**, PC3 **(E)**, and MDA-MB-231 **(F)**. The PBS control and the two negative control antibodies (Her2-13C1 and aVE201) were also included.

Expression of the apoptosis-related proteins was also examined by flow cytometry. Expression of Bax (**Figures 5A–C**), c-caspase-3 (**Figures 6A–C**), and c-PARP-1 (**Figures 6D–F**) was up-regulated, and Bcl-2 expression (**Figures 5D–F**) was down-regulated after DU145, PC3 and MDA-MB-231 cells were treated with the three anti-CXCR4 scFvs compared to PBS control and two negative control antibodies (Her2-13C1 and aVE201). These results suggest that the three anti-CXCR4 scFvs can induce cell apoptosis in these three cancer cell lines.

### Induction of Cancer Cell Apoptosis by scFvs Was Detected by Western Blot Analysis

**Figure 7:**
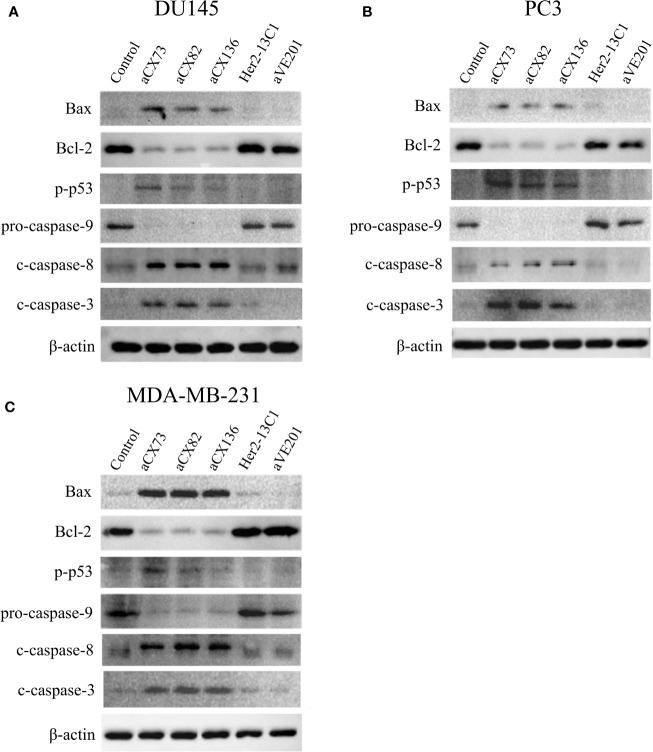
Expression of the apoptosis-related proteins was examined by Western blot analysis. Cells were treated for 48 h with anti-CXCR4 scFvs (aCX73, aCX82, and aCX136), negative control antibodies (Her2-13C1 and aVE201), or PBS as a control. Effect of the antibodies on the expression of the apoptosis-related proteins was tested in DU145 **(A)**, PC3 **(B)**, and MDA-MB-231 **(C)**. β -actin was used as an internal control. Western blots were analyzed using ImageJ software.

To further study effect of the anti-CXCR4 scFvs on cell apoptosis, the expression of the apoptosis-related proteins was analyzed by Western blot analysis. Results showed that anti-CXCR4 scFvs induced the expression of the pro-apoptotic proteins Bax, p-p53, c-caspase-8, and c-caspase-3 and decreased the expression of Bcl-2 and pro-caspase-9 in all three cancer cell lines compared to the PBS control (**Figure 7**). Anti-CXCR4 scFvs increased the Bax/Bcl-2 ratio in all three cancer cell lines. In DU145 cells, Bax/Bcl-2 ratio increased ranging from 7.17 to 11.23 compared to the PBS control (1.0). In PC3 cells, Bax/Bcl-2 ratio increased ranging from 12.69 to 20.72 compared to the PBS control (1.0). In MDA-MB-231 cells, Bax/Bcl-2 ratio increased ranging from 16.93 to 21.92 compared to the PBS control (1.0). The two negative control antibodies (Her2-13C1 and aVE201) did not affect the expression of these proteins compared to the PBS control. These results confirmed that the three anti-CXCR4 scFvs could induce cell apoptosis in these three cancer cell lines.

### scFvs Inhibit the Migration and Invasion of Cancer Cells

**Figure 8:**
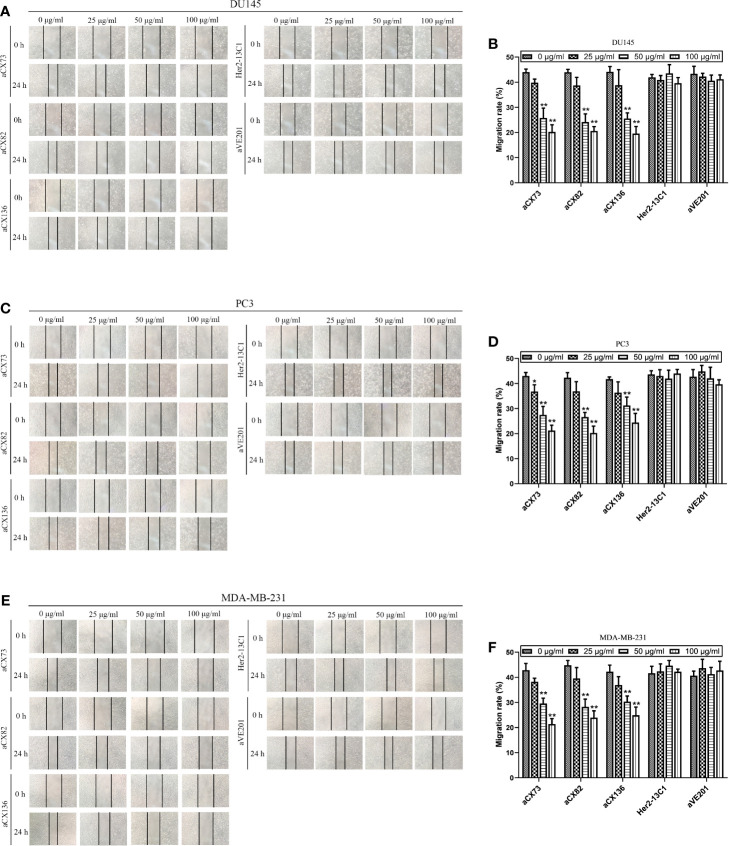
Anti-CXCR4 scFvs can decrease cell migration detected by wound healing assay. Cells were cultured with CXCL12 (100 ng/ml) and the three anti-CXCR4 scFvs (aCX73, aCX82, and aCX136) or negative control antibodies (Her2-13C1 and aVE201). Images were taken at 0 and 24 h after the cell wounds were made. The wound widths were determined. **(A, B)** DU145; **(C, D)** PC3; **(E, F)** MDA-MB-231. The results represent mean ± S.D (n=5). *P < 0.05 and **P < 0.01.

**Figure 9:**
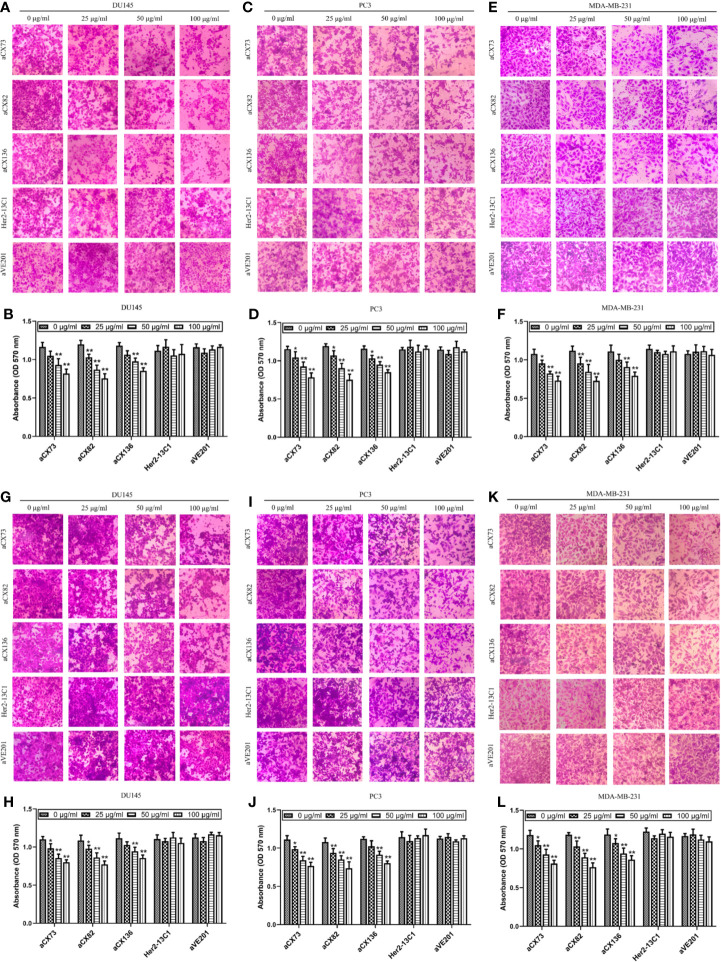
Anti-CXCR4 scFvs can decrease cancer cell migration and invasion. To detect cell migration **(A–F)**, cells were incubated with CXCL12 (100 ng/ml) and the anti-CXCR4 scFvs (aCX73, aCX82, and aCX136) or two negative control antibodies. The cells that crossed over the membrane were treated with crystal violet, which was solubilized in acetic acid. Optical density was detected. **(A, B)** DU145; **(C, D)** PC3; **(E, F)** MDA-MB-231. For the detection of cell invasion, matrigel was placed in transwells, and then, cells were added. **(G, H)** DU145; **(I, J)** PC3; **(K, L)** MDA-MB-231. The results represent mean ± S.D (n = 5). *P < 0.05 and **P < 0.01.

Wound healing assay was performed to examine effect of the three scFvs on the migration of cancer cells. The three scFvs inhibited DU145, PC3, and MDA-MB-231 cell migration in a dose-dependent manner by wound healing assay (**Figure 8**). In contrast, the two negative control antibodies (Her2-13C1 and aVE201) had no effect on the migration of these cells. Transwell assay was also performed to examine effect of the three scFvs on the migration of cancer cells. The similar results were seen in transwell assay (**Figures 9A–F**). These results suggested that the three scFvs could decrease the migration of these cancer cells.

Effect of the three scFvs on the invasion of cancer cells was also tested by transwell invasion assay with the matrigel-coated transwells. The three scFvs inhibited DU145 invasion (**Figures 9G, H**). In contrast, the two negative control antibodies (Her2-13C1 and aVE201) did not affect the DU145 invasion. The same results were seen for PC3 (**Figures 9I, J**) and MDA-MB-231 (**Figures 9K, L**). These results suggested that the three scFvs could inhibit the invasion of these cancer cells.

### scFvs Decrease Tumor Growth in a Xenograft Model

**Figure 10:**
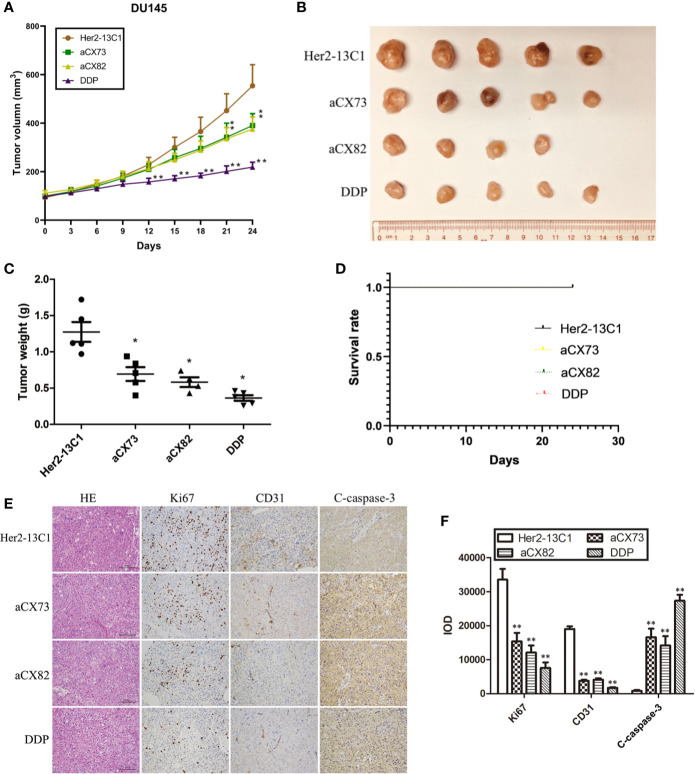
Anti-CXCR4 scFvs can decrease DU145 xenograft growth. **(A)** Mice were inoculated with DU145 cells subcutaneously. Following tumors formation, mice were inoculated intravenously with the anti-CXCR4 scFvs (aCX73 and aCX82), the negative control antibody Her2-13C1 and DDP. **(B, C)** After euthanization of the mice on day 24 after the first inoculation, the tumors were excised. **(D)** The survival rates of mice treated with aCX73, aCX82, and DDP were determined compared to the negative control antibody Her2-13C1 using a log-rank (Mantel-Cox) test. Only the black line for the first group (Her2-13C1) was seen, and the other color lines for the other groups were invisible because all the color lines for all the groups were in the exact same position. **(E)** Tumor sections were incubated with HE or antibodies against Ki67, CD31 and c-caspase-3. **(F)** IOD was determined from **Figure 10E**. Results represent mean ± S.D. *P < 0.05 and **P < 0.01.

Animal study was performed to examine the effect of the scFvs on the inhibition of tumor growth in DU145 xenograft model. Mice were administrated with anti-CXCR4 scFvs (aCX73 and aCX82, 10 mg/kg), the negative control antibody (Her2-13C1, 10 mg/kg) and DDP (2 mg/kg) as a positive control when the tumor volumes were about 100 mm^3^. On day 21 following the first injection of the test reagents, aCX73 (average tumor volume 342.1 mm^3^, *P* < 0.05) and aCX82 (average 336.1 mm^3^, *P* < 0.05) achieved a statistically significant reduction of tumor volumes compared to the negative control antibody Her2-13C1 (average 451.6 mm^3^). On day 24 following the first injection of the test reagents, aCX73 (average 390.1 mm^3^, *P* < 0.05) and aCX82 (average 375.3 mm^3^, *P* < 0.05) also showed a statistically significant reduction of tumor volumes compared to the negative control antibody Her2-13C1 (average 554.4 mm^3^) (**Figures 10A, B**). On day 24 following the first injection of the test reagents, all the mice were sacrificed and examined by the naked eye, and no clear gross anatomical change in organs including livers, kidneys, hearts, lungs and spleens and the other parts of bodies was observed from the mice treated with the three scFvs and the other test reagents, and no behavioral abnormalities or any color change in any parts of bodies were observed. This observation suggests that these scFvs may not induce obvious adverse side or toxic effects in mice and needs to be confirmed by blood tests on the mice analyzing the organ parameters and microscopic examination of the organs. The mice treated with these scFvs did not show the adverse side or toxic effects, such as diarrhea, fatigue and low food and water consumption.

On day 24, the tumor weight was also significantly lower in mice treated with the scFvs compared to the negative control antibody (**Figure 10C**). DDP as a positive control also showed a statistically significant tumor weight reduction compared to the negative control antibody. Each of Her2-13C1, aCX73 and DDP groups included five mice. The aCX82 group included four mice because only four mice containing about 100 mm^3^ tumor volumes were available for the first injection of this scFv. On day 24 following the first injection of the test reagents, all mice survived, and there was no difference in median survival time of mice among all the groups (**Figure 10D**). There was no difference in the risk of developing tumors in mice (hazard ratio) among all the groups (**Table 1**).

**Table 1:** The risk of developing tumors in mice (hazard ratio) treated with the scFvs and DDP compared to the negative control antibody Her2-13C1 using a log-rank (Mantel-Cox) test.

Group	N	HR	P value
aCX73	5	Undefined	>0.9999
aCX82	4	Undefined	>0.9999
DDP	5	Undefined	>0.9999

N, the number of mice; HR, hazard ratio.

The tumor sections were stained with HE or antibodies against Ki67, CD31, and c-caspase-3, and the integral optical density (IOD) was calculated. Immunohistochemistry analysis of tumors showed a significantly decreased Ki67 and CD31 expression and a significantly increased c-caspase-3 expression in mice treated with the scFvs compared to the negative control antibody Her2-13C1 (**Figures 10E, F**), suggesting that the two scFvs significantly decreased cancer cell proliferation and tumor angiogenesis and increased cell apoptosis. These results suggested that the two anti-CXCR4 scFvs (aCX73 and aCX82) could inhibit the tumor growth in DU145 xenograft model.

## Discussion

CXCR4 is over-expressed in different cancer cells and is considered as a prognostic marker of various cancers including breast cancer (31), ovarian cancer (32), leukemia (33), prostate cancer (34), and colorectal cancer (35). Moreover, CXCL12/CXCR4 axis plays an important role in tumor growth and metastasis. Therefore, cancer treatment by targeting CXCR4 signaling pathways should be beneficial (15). Some cancer therapeutics was developed to target CXCR4/CXCL12 signaling pathways. AMD3100 is a small molecule CXCR4 antagonist and can inhibit CXCL12/CXCR4 signaling pathways. Studies showed that AMD3100 inhibited metastasis in animal models for ovarian and colorectal cancers, melanoma, and oral squamous cell carcinoma (36–41). mAbs, such as BMS-936564/MDX-1338 and LY2624587, could also suppress CXCR4/CXCL12 signaling pathways and inhibit tumor growth and metastasis in different animal models both *in vitro* and *in vivo* (42, 43).

Chemokine receptors including CXCR4 represent a sub-class of G protein-coupled receptors (GPCRs) containing seven transmembrane segments. Similar to other GPCRs, CXCR4 has an extracellular N-terminus, seven transmembrane alpha helices connected by three extracellular loops (ECL) and three intracellular loops (ICL) and a C-terminus that is located in the cytoplasm. In addition to the disulfide bond between CXCR4 helix VII (Cys 274) and the N-terminal region (Cys 28), the disulfide bond between CXCR4 ECL2 (Cys 186) and the extracellular end of alpha helix III (Cys 109) is essential for its ligand CXCL12 binding to CXCR4 by shaping the entrance to the ligand-binding pocket, and is highly conserved among the chemokine receptors (44, 45). Studies also showed that only the peptides derived from ECL2 were able to specifically bind to CXCL12 and inhibit its interactions with CXCR4 thereby preventing receptor activation (46). So, ECL2 is most likely to be the major determinant of the CXCR4 Chemokine Recognition Site 2 (CRS2) (46). In this study, the CXCR4 ECL2 was selected as an antigen for screening the human scFv antibody library. It may contribute to the anti-tumor effects of the anti-CXCR4 scFvs cloned in this study.

Human scFv antibody has the following advantages compared to the conventional mAb. It can easily penetrate into tumor tissue due to its small size and contains no antigenicity. It can greatly reduce the cost to be manufactured because it can be produced in *E. coli*. In addition, scFv can be modified to further increase its binding to antigen and its efficacy for cancer therapy (47). After two anti-CD123 scFvs were linked to form a bivalent scFv (biscFv), the overall efficacy of the anti-CD123 biscFv in binding to CD123 and inhibiting the binding of CD123 to its ligand in TF-1 cells was significantly increased (48). An anti-EGFR scFv was fused to the plant toxin gelonin (rGel) to get a targeted toxin, which could selectively bind EGFR-overexpressing cells with high affinity and significantly suppressed the tumor growth of human NSCLC (49). Some anti-tumor small molecular drugs have no tumor specificity and high toxicity to normal tissues (50). To overcome this problem, a study showed that nanoparticle surfaces were covered with anti-CD44v6 scFvs, and arsenite ion (As) as the anti-cancer drug was encapsulated inside the nanoparticles, thus providing a safe and targeted carrier for drug delivery. When the modified nanoparticles entered tumor cells by endocytosis, they were rapidly degraded, and arsenite ion was released into cytoplasm and induced cell apoptosis (51, 52). Therefore, scFv can be modified to increase its binding to its antigen and enhance its efficacy for cancer therapy.

In this study, three anti-CXCR4 scFvs were isolated by the yeast two-hybrid system, which is based on the GAL4 protein properties of saccharomyces cerevisiae (27). Yeast two-hybrid system offers many advantages, such as high sensitivity, convenience, and cost-saving. However, it has a problem with getting false-positive clones. To solve this problem, two reporter genes (His and Ade) were used to provide strong nutrition screening and eliminate a large number of false-positive clones. Trp^+^ bait plasmid and the Leu^+^ scFv library plasmid were used to increase the number of positive clones. In addition, 3-AT can inhibit the background His expression and was added to SD/-Trp/-Leu/-His/-Ade plates to effectively eliminate false positive clones (53). The empty plasmid pGBKT7 containing no additional protein and pGBKT7-X (X as an unrelated protein) were also included as controls in the library screening process. In this study, three anti-CXCR4 scFvs were obtained from the human scFv library by yeast two-hybrid system, and they showed anti-tumor effect *in vitro* and *in vivo*.

This study showed that anti-CXCR4 scFvs induced the expression of the pro-apoptotic proteins Bax, p-p53, c-caspase-8, c-caspase-3, and c-PARP-1 and decreased the expression of Bcl-2 and pro-caspase-9 in all three cancer cell lines compared to the two negative control antibodies (**Figures 5**–**7**). BAX is a member of the Bcl-2 gene family and promotes apoptosis by binding to and antagonizing Bcl-2 protein (54, 55). The p53 is a transcription factor that, when activated as part of the cell response to stress, regulates many downstream target genes including BAX. Caspases play an important role in the regulation of apoptosis signaling pathway. Caspase-8 and 9 are the upstream initiator caspases which can trigger the activation of the downstream executioner caspases including caspases 3, 6, and 7. These activated caspases cleave various cellular substrates and eventually lead to cell apoptosis (56, 57). As detected by flow cytometry, the induction of the pro-apoptotic proteins (Bax, c-caspase-3, and c-PARP-1) by the three scFvs was modest, ranging from 12.7 to 32.4% compared to the two negative control antibodies, ranging from 2.83 to 6.35% (**Figures 5** and **6**), while the results of Western blot analysis were more convincing than flow cytometry (**Figure 7**). This study demonstrates that the three anti-CXCR4 scFvs can induce cell apoptosis of the three cancer cell lines by the regulation of apoptosis signaling pathway.

## Data Availability Statement

The datasets presented in this study can be found in online repositories. The names of the repository/repositories and accession number(s) can be found below: https://www.ebi.ac.uk/ena, LR743525 https://www.ebi.ac.uk/ena, LR743526 https://www.ebi.ac.uk/ena, LR743527.

## Ethics Statement

The animal study was reviewed and approved by the Institutional Animal Care and Use Committee of Jinan University.

## Author Contributions

HW and GC contributed to the conception and design of the study. G-QL, JL, X-XZ, Z-XL, and TC performed the experiments, analyzed the data, and drafted the manuscript. All authors contributed to manuscript revision and read and approved the submitted version.

## Funding

This work was supported by fund from the Science and Technology Program of Guangdong No. 2017A020211014 (H. Wei).

## Conflict of Interest

The authors declare that the research was conducted in the absence of any commercial or financial relationships that could be construed as a potential conflict of interest.
